# Predicting cognitive dysfunction and regional hubs using Braak staging amyloid-beta biomarkers and machine learning

**DOI:** 10.1186/s40708-023-00213-8

**Published:** 2023-12-03

**Authors:** Puskar Bhattarai, Ahmed Taha, Bhavin Soni, Deepa S. Thakuri, Erin Ritter, Ganesh B. Chand

**Affiliations:** 1 Department of Radiology, Mallinckrodt Institute of Radiology, Washington University School of Medicine, St. Louis, MO USA; 2 University of Missouri School of Medicine, Columbia, MO USA; 3 Department of Biomedical Engineering, Washington University McKelvey School of Engineering, St. Louis, MO USA; 4 Imaging Core, Knight Alzheimer Disease Research Center, Washington University School of Medicine, St. Louis, MO USA; 5 Institute of Clinical and Translational Sciences, Washington University School of Medicine, St. Louis, MO USA; 6 NeuroGenomics and Informatics Center, Washington University School of Medicine, St. Louis, MO USA

**Keywords:** Mild cognitive impairment, Machine learning, Amyloid-beta, Feature importance, Braak staging, Neuroimaging

## Abstract

Mild cognitive impairment (MCI) is a transitional stage between normal aging and early Alzheimer’s disease (AD). The presence of extracellular amyloid-beta (Aβ) in Braak regions suggests a connection with cognitive dysfunction in MCI/AD. Investigating the multivariate predictive relationships between regional Aβ biomarkers and cognitive function can aid in the early detection and prevention of AD. We introduced machine learning approaches to estimate cognitive dysfunction from regional Aβ biomarkers and identify the Aβ-related dominant brain regions involved with cognitive impairment. We employed Aβ biomarkers and cognitive measurements from the same individuals to train support vector regression (SVR) and artificial neural network (ANN) models and predict cognitive performance solely based on Aβ biomarkers on the test set. To identify Aβ-related dominant brain regions involved in cognitive prediction, we built the local interpretable model-agnostic explanations (LIME) model. We found elevated Aβ in MCI compared to controls and a stronger correlation between Aβ and cognition, particularly in Braak stages III–IV and V–VII (*p* < 0.05) biomarkers. Both SVR and ANN, especially ANN, showed strong predictive relationships between regional Aβ biomarkers and cognitive impairment (*p* < 0.05). LIME integrated with ANN showed that the parahippocampal gyrus, inferior temporal gyrus, and hippocampus were the most decisive Braak regions for predicting cognitive decline. Consistent with previous findings, this new approach suggests relationships between Aβ biomarkers and cognitive impairment. The proposed analytical framework can estimate cognitive impairment from Braak staging Aβ biomarkers and delineate the dominant brain regions collectively involved in AD pathophysiology.

## Introduction

Alzheimer’s disease (AD) is a progressive illness that can start with no noticeable symptoms and advance to severe symptomatic forms [1, 2]. Mild cognitive impairment (MCI) is considered a transitional state between normal aging cognitive changes and early AD [3]. Amyloid-beta (Aβ) and tau are two vital hallmarks of AD, but their relationship with MCI is poorly understood. Therefore, regional Aβ biomarkers based on the tau-defined topological regions and their multivariate predictive relationships with cognitive impairment are yet to be discovered.

The amyloid cascade hypothesis in AD [4, 5] postulates that the accumulation of Aβ plaques is the primary event that leads to a sequence of intracellular neurofibrillary tangle accumulation, synaptic dysfunction, and gliosis, eventually resulting in symptomatic AD dementia in later stages of the disease [5, 6]. However, Aβ accumulation could be the crucial step in a more complicated pathophysiological process [7]. AD diagnosis has shifted from postmortem histopathology to PET imaging using Aβ radiotracers, such as Pittsburgh Compound B (PiB) [8, 9]. Aβ accumulation occurring decades before the onset of clinical symptoms in MCI and mild/moderate AD [10, 11] has driven the search for novel biomarkers [12, 13]. Braak staging is a method of classifying tau pathology in AD, with six Braak stages: I–II, III–IV, and V–VI representing the progression of tau accumulation [14, 15].

The association between regional tau accumulation and cognitive decline is somewhat established [16–21]. However, the accumulation of Aβ plaques in the topographic map of Braak staging in the MCI population remains unknown. Although the temporal relationship between tau and Aβ accumulation is somewhat debatable, many studies suggest that Aβ biomarkers may present very early during illness. Jack et al. [22] suggested that the shift from Aβ+/tau− to Aβ+/tau+ is linked to severe cognitive decline; however, the connection between global/regional tau deposition topography and Aβ is still unknown [23]. Several studies [14, 15, 24, 25] suggest that AD pathology develops in the Braak stages more dominantly and worsens significantly during the illness course. We thus selected regional Aβ biomarkers from these Braak regions to estimate cognitive decline. Specifically, it is unclear whether regional Aβ measures have multivariate relationships with cognitive impairment and whether such relationships can be predicted using advanced machine learning (ML) algorithms. In this study, we chose amyloid PET over tau PET, because prior studies [26] suggest that amyloid biomarkers start to appear much earlier compared to tau biomarkers in the AD continuum, and we seek to characterize the multivariate relationships between those early biomarkers and cognition using ML.

Herein, we propose machine learning approaches that seek to estimate the multivariate predictive relationships between Aβ biomarkers and cognitive impairment. We hypothesized that cognitive dysfunction can be estimated from regional Aβ biomarkers via ML modeling methods. We further hypothesized that the Aβ-related dominant brain regions involved with such cognitive processes can be identified by integrating feature importance and predictive modeling methods.

## Materials and methods

### Participants

We used data from the OASIS database [27] as per our previous work for participant selection and image processing [28, 29]. Written informed consent was obtained from all participants in this OASIS study following the institutional review board procedure at Washington University in St. Louis. We used only those who underwent dynamic imaging of PiB for DVR analysis and selected only 60 participants who met our in-house quality control criteria. Subject demographics are shown in Table 1. We included only AD-related MCI participants, as previously described [28, 30]. Individuals with other conditions such as vascular dementia, primary progressive aphasia, major depression, a history of clinically significant stroke, active neurologic or psychiatric illness, abnormal MRIs, and those using psychoactive drugs were excluded from this study [28, 30]. The clinical dementia rating (CDR) scale was used to assess cognitive and functional abilities. In our study, MCI had a CDR [31, 32] of 0.5–1, as well as a memory box score of 0.5 or greater, while controls had a CDR of 0 and a memory box score of 0.

**Table 1:** Summary of demographics of participants

	MCI	HC
Total (*N*)	33	27
Average MMSE (SD)	26.70 (2.64)	28.70 (1.64)
Females (*N*)	15	15
Males (*N*)	18	12
Age in years (SD)	77.3 (6.68)	73.3 (7.48)
Education in years (SD)	16 (3.06)	15.29 (2.26)

The mini-mental state examination (MMSE) [33] is the most widely used cognitive test to diagnose MCI [33, 34] and rule out dementia [35, 36]. It also predicts the prognosis from MCI to AD [37]. The MMSE has a maximum score of 30, and scoring 23 or less indicates cognitive impairment [33, 38]. A higher risk of mortality is linked with a lower MMSE [39]. We employed the MMSE as a metric for cognitive abilities and as an output variable.

### Data acquisition and processing

Data acquisition and processing were performed as per our previous work [28, 29]. Briefly, a bolus injection of [11]C-PiB was followed by Dynamic PET 3D scans that were acquired for more than an hour using the ECATHR plus 962 PET scanner or the Biograph 40 PET/CT scanner. All participants got T1-weighted brain MRI using 3T Biograph MR or 1.5T Vision, Siemens 3T TrioTim. Multi-atlas region Segmentation utilizing Ensembles of registration algorithms and parameters and locally optimal atlas selection (MUSE) was used for individual T1-MRI segmentation [28, 29, 40–42]. We quantified dynamic PET scans as distribution volume ratio (DVR) outcomes [43]. To account for partial volume effects [44], we applied a parallel level set (PLS) regularization-based partial volume correction method [45]. We leveraged our previously developed and validated harmonization approach [28, 29, 40] to harmonize multi-scanner data sets. We used data from all study participants to compute coefficients accounting for the effect of factors such as site, age, and sex and applied them to each participant [28, 29]. After correcting for these factors, we generated harmonized DVRs of Braak staging regions (Table 2) and used them in all analyses.

**Table 2:** Brain regions of different Braak stages used in our study (GM: grey matter)

Braak stages	Brain regions
I–II	• Hippocampus and Entorhinal cortex
III–IV	• Parahippocampal gyrus, Fusiform gyrus, Occipital fusiform gyrus, Lingual gyrus, Amygdala, Inferior temporal gyrus, Middle temporal gyrus, Temporal pole, Cingulate gyrus, and Insula
V–VI	• Inferior frontal GM, Lateral frontal GM, Medial frontal GM, Opercular frontal GM, Parietal GM, Supratemporal GM, Superior temporal gyrus, Lateral occipital GM, Cuneus, and Calcarine cortex

### ML-based predictive modeling algorithms

**Fig. 1:**
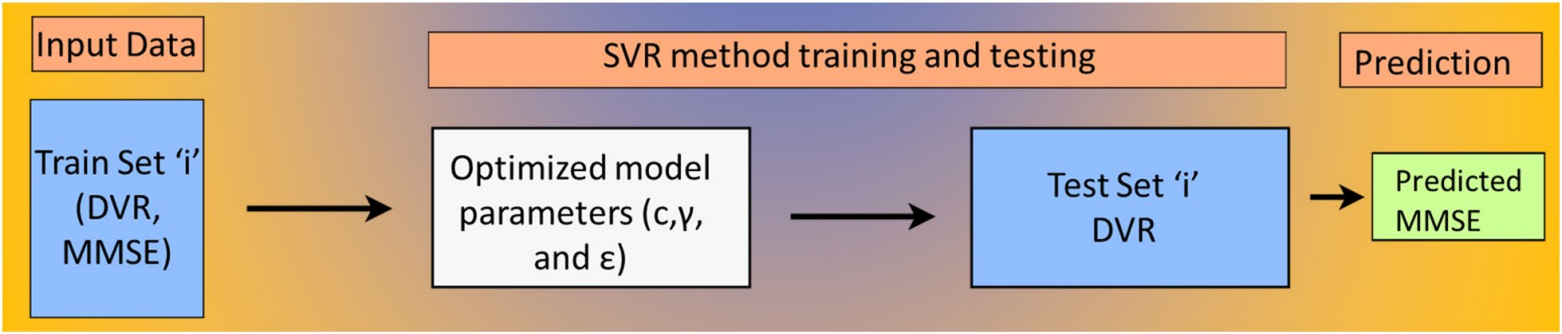
SVR model predicts MMSE with DVRs as feature variables. A tenfold CV was applied to the DVR and MMSE data sets. Grid Search CV is used for determining the optimum *c*, *ϵ*, and *γ* values in each fold. The SVR model used these values to fit train data and predict test set MMSE outcomes. The process is repeated for all tenfold data combinations

This study used two ML techniques, SVR [46–48] and deep learning [49, 50] based ANN [51, 52] to predict MMSE. The SVR model, consistent with previous research [28, 29, 40, 41], was created using the sci-kit-learn package [53, 54] in Python. We employed tenfold cross-validation (CV), where the data set was divided into ten subsets, and on each fold, the model was trained on those nine subsets of data and was tested on an entirely unseen subset. Hence, we can argue that these models are generalizable and can be used to train the biomarkers of the brain regions to predict cognitive decline. During each fold, the training and test sets were preprocessed using standard scaling, with the scaler fitted to the training set before being transformed and applied to the test data. Standard scaler standardizes feature values by finding the z scores corresponding to each feature value using $z=\frac{\left(x-\mu \right)}{s}$, where x represents the feature value; $\mu$ and s represent the mean and standard deviation of the samples. The transformed training set was then fitted to the SVR model (Fig. 1) with a radial basis function (RBF) kernel [55] using optimal parameters obtained by Grid Search CV from the pool of the following hyperparameters:

*C* values: 2^*n*^, *n* = − 5, − 4, − 3, …, 13, 14

Gamma: 2^*n*^, *n* = − 12, − 11, …, 1, 2

Epsilon: 2^*n*^, *n* = − 7, − 6, …, − 1, 0

The model was optimized using mean absolute error (MAE) as the loss function, which was then used to predict the MMSE. The same SVR model designs were applied to each Braak stage and their combinations.

**Fig. 2:**
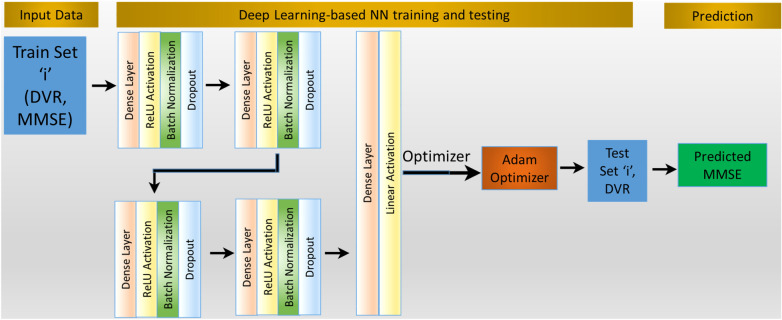
ANN model predicts MMSE with DVRs as feature variables through a tenfold CV. Batch normalization and dropout are applied after each dense layer and ReLU activation, except for the output layer. Input dimension shape was used for the first dense layer with 40 units. The next three hidden layers contain 30, 20, and 10 units. The final output layer contains only one unit with a linear activation function for the regression problem

We used a consistent tenfold CV approach with standard scaling for the ANN model to predict the MMSE score. This ANN sequential model has four dense layers, each with a random normal kernel initializer, and was implemented on Python using TensorFlow [56] and Keras [57] with a dropout of 0.5 and batch normalization [58, 59] deployed after the input and all hidden dense layers [49]. The input layer has 40 units, and the subsequent three hidden dense layers have 30, 20, and 10 units with a rectified linear activation unit (ReLU) [60–62] as the activation function. Our regression model uses a linear activation function in the output layer (Fig. 2**)**. This model was trained and optimized on the training data for each fold using the Adam optimizer [63] and MAE as the loss function, with the starting learning rate within a range of 0.001 to 0.05 with a step of 0.001, and chose the results and the feature importance values corresponding to the learning rate that yielded the maximum correlation between the actual and predicted MMSE. We used a batch size of 6 and a validation split of 0.05 to monitor the model’s performance while training and avoid overfitting. An early stopping callback was used to stop training when the loss function reached a plateau and restore ideal weights. Identical ANN models were used for MMSE prediction on each Braak stage and its combination. All the plots used in this paper were plotted using Matplotlib [64] and Seaborn [65] in Python.

It is a challenging task to understand the predictions made by SVR/ANN as the internal mechanisms of these models are complex, non-intuitive, and less interpretable. However, neuroimaging data sets and the development of ML have made it possible to identify areas that are strongly associated with a specific trait or feature [66, 67]. Feature importance measures determine the significance of specific features for a given model by quantifying how much performance changes when a particular feature is randomly shuffled. Local Interpretable Model-Agnostic Explanations (LIME) [68] is one of the most comprehensive methods for feature importance [69]. It interprets which features contributed significantly while predicting the MMSE, providing insights into the relationship between the DVR values of the brain regions and the MMSE. It estimates the feature significance by approximating the predictions of a complex model with a simple interpretable model and accounts for interactions between the features. The feature importance with LIME is calculated using the LIME package in Python [70].

The LIME technique interprets predictions of a complex ML model f with inputs x by locally approximating the model in the vicinity of the prediction. This simple explainable model follows the additive feature attribution [71] given by the following equation:

1 $$g\left( {z^{\prime}} \right) = \phi_{0} + \mathop \sum \limits_{i = 1}^{M} \phi_{i} z_{i}{\prime}$$

where ${z}{\prime}$ represents the non-zero components of perturbed input features z in the explanation model $g \epsilon G$, where G is a class of potentially interpretable models, such that $z \epsilon {\left\{\mathrm{0,1}\right\}}^{M}$, M is the number of simplified input features and ${\phi }_{i}$ representing the contribution of each feature to the output, and the sum of these weighted contributions produces the final output of the model.

LIME is obtained by minimizing the function given by the following equation:

2 $$\xi = \begin{array}{*{20}c} {\arg min} \\ {g \in G} \\ \end{array} L\left( {f,g, \pi_{x} } \right) + {\Omega }\left( g \right)$$

where $L\left(f,g, {\pi }_{x}\right)$ evaluates local fidelity by determining how inaccurately g approximates f in the locality defined by ${\pi }_{x}$, ${\pi }_{x}$ being a measure of proximity between an instance z to x and $\Omega \left(g\right)$ measures the complexity of the simplified model that penalizes models that are too complex by adding a regularization term to the optimization function.

In each fold of CV, the LIME explainer was trained in regression mode with ‘lasso_path’ [54] as the ‘feature_selection’ parameter using the train data. The ‘lasso_path’ approach is often recommended for addressing the problem of highly correlated features, because it uses L1 regularization, which can mitigate multicollinearity by restricting the sum of the absolute values of the coefficients of the features [72]. LIME values were then generated for each instance of the test set for all features using Lasso as the ‘modelregressor’. While explaining, we used ‘num_features = 22’ with a maximum iteration of 1000, a tolerance of 0.1, and the best value of alpha. The best value of α, which controls the strength of regularization in the regressor, was found using ‘Grid Search CV’ from the scikit-learn library within [0.01, 0.05, 0.1, 0.2, 0.5, 1, 2, 10] using Lasso as the estimator and MAE as the scoring hyperparameter. The mean of the absolute LIME values was accessed to determine the importance of each feature. The Lasso regressor includes a penalty term in its objective function that encourages sparsity in the selected features, resulting in only the most important features being shrunk to a lesser extent, while the less important features are set closer to zero. This approach can enhance the model's predictive performance by reducing overfitting and producing more interpretable features.

LIME generates the interpretable model by creating perturbed versions of the original data and training a model on the new data. Although LIME is one of the most stable methods [73, 74], due to the random nature of the sampling procedure to generate the perturbed versions, executing LIME more than once might sometimes result in various interpretable models and hence different feature significance values depending on the data and the model used [69, 75, 76]. Hence, to obtain even more consistent feature significance values as a precautionary approach, we ran the feature importance technique three times with ten folds of CV, averaging the feature importance values over these repetitions. CV for the feature importance methods [77] can help reduce the impact of random sampling variability in LIME.

### Statistical analysis

We used the Mann–Whitney *U* test [78] to compare DVR between control and MCI in each Braak stage. Spearman correlation along with associated *p* value was used to examine the relationship between MMSE and DVR and to determine the predictive associations between actual MMSE and predicted MMSE. These values were calculated using the scipy [79] library in Python. To adjust the statistical significance for multiple comparisons, we used a false discovery rate (FDR) [80] employing ‘multipletests’ from ‘statsmodels.stats.multitest’ in the Python library using the method ‘fdr_bh’.

## Results

### DVR comparison between MCI and controls

**Fig. 3:**
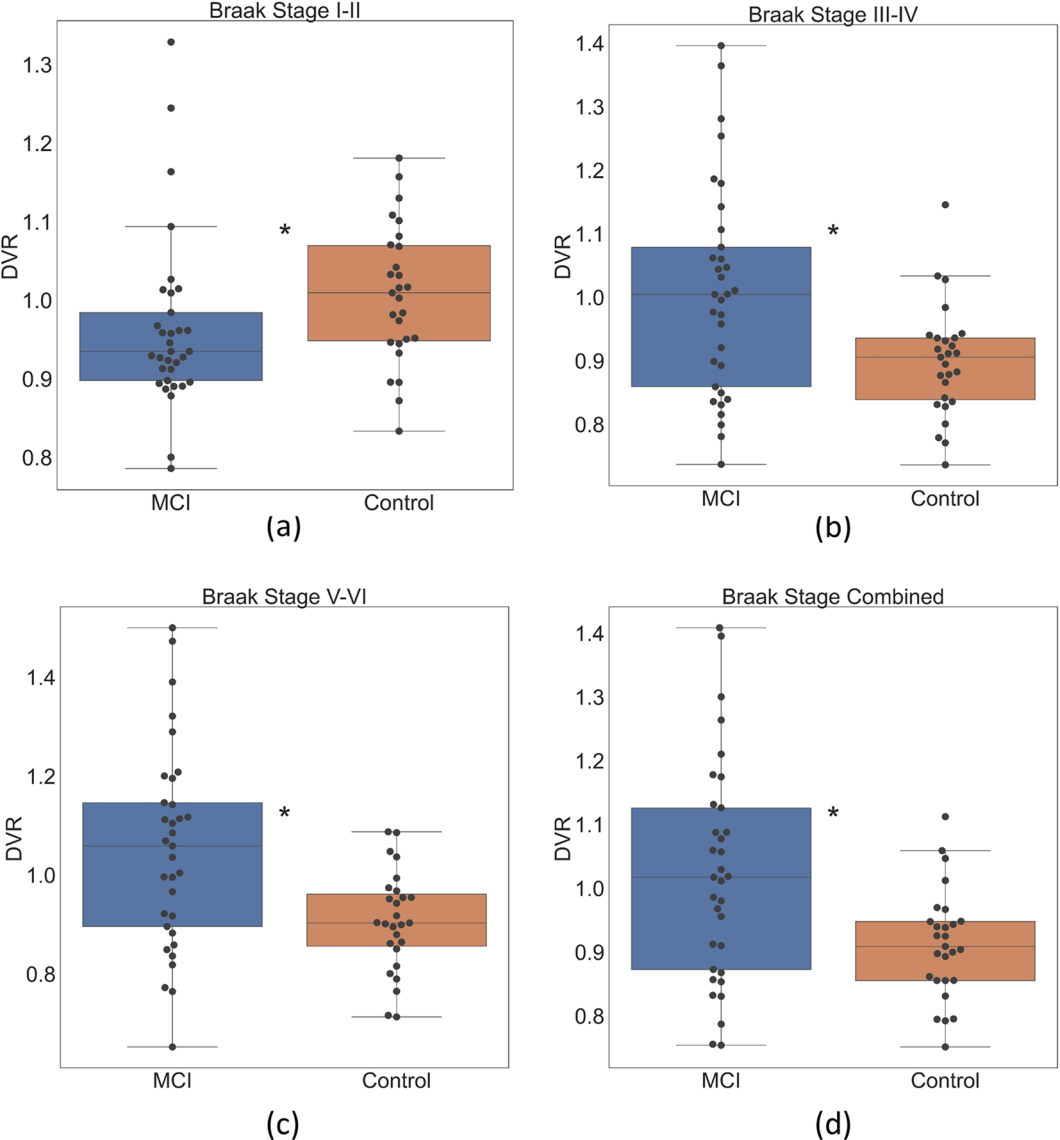
Boxplots and scatterplots of Aβ DVR comparison between mild cognitive impairment (MCI) and controls in different Braak stages (*: FDR-*p* < 0.05)

Figure 3 shows a DVR comparison between MCI and controls within each Braak stage. Compared to controls, MCI had lower DVR in Braak stages I–II (FDR-*p* = 0.012). Compared to controls, MCI had higher DVR in Braak stages III–IV (FDR-*p* = 0.008), Braak stages V–VI (FDR-*p* = 0.002), and all stages combined (FDR-*p* = 0.007).

### Associations between DVR and MMSE

**Fig. 4:**
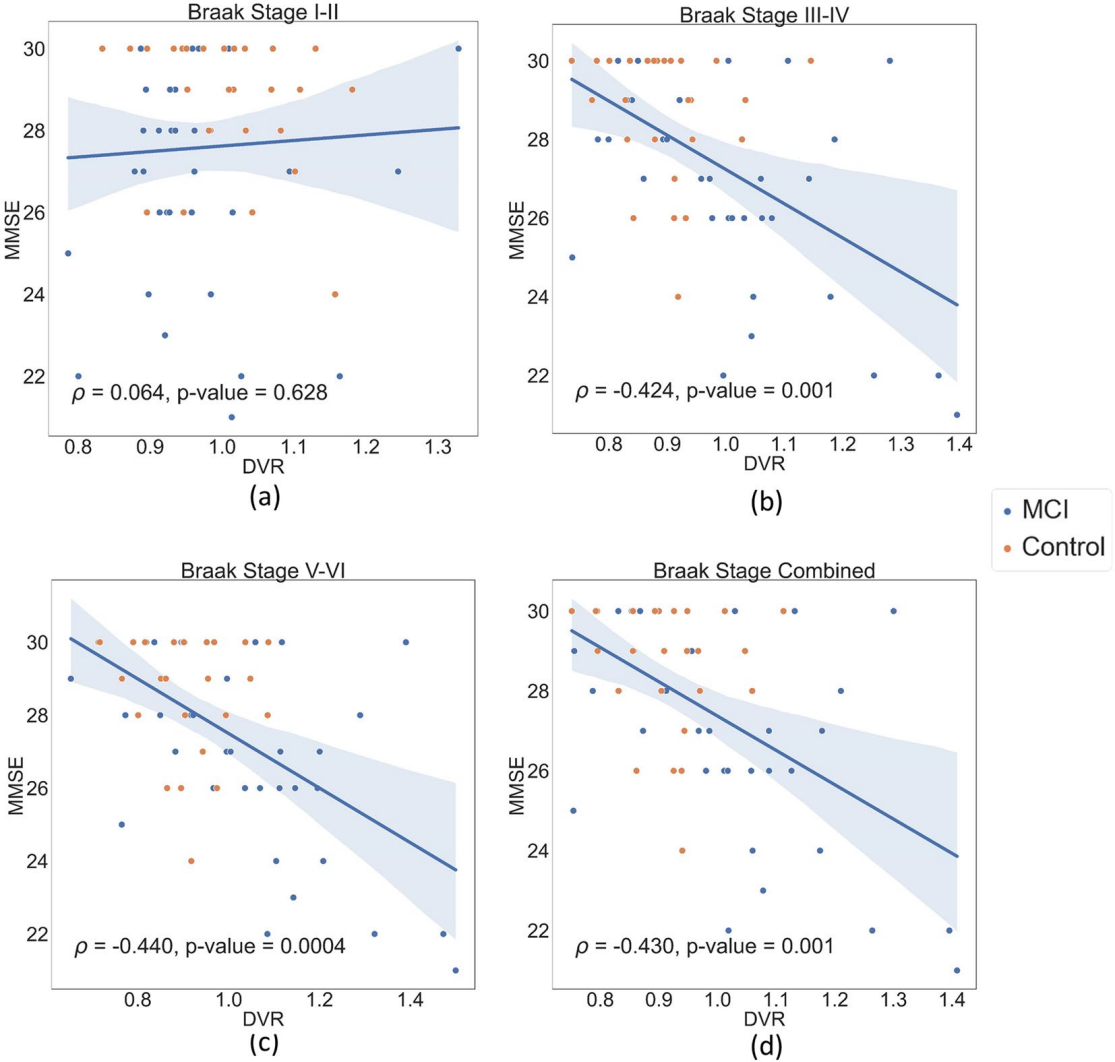
DVR of various Braak stages correlates with MMSE in MCI and controls. **a** Has a weak positive correlation for Braak I–II, while (**b**–**d**) have a negative correlation for III–IV, V–VI, and all stages combined (FDR-*p* < 0.05). Linear fit (solid line) and 95% confidence interval (shadowed area) are shown

**Fig. 5:**
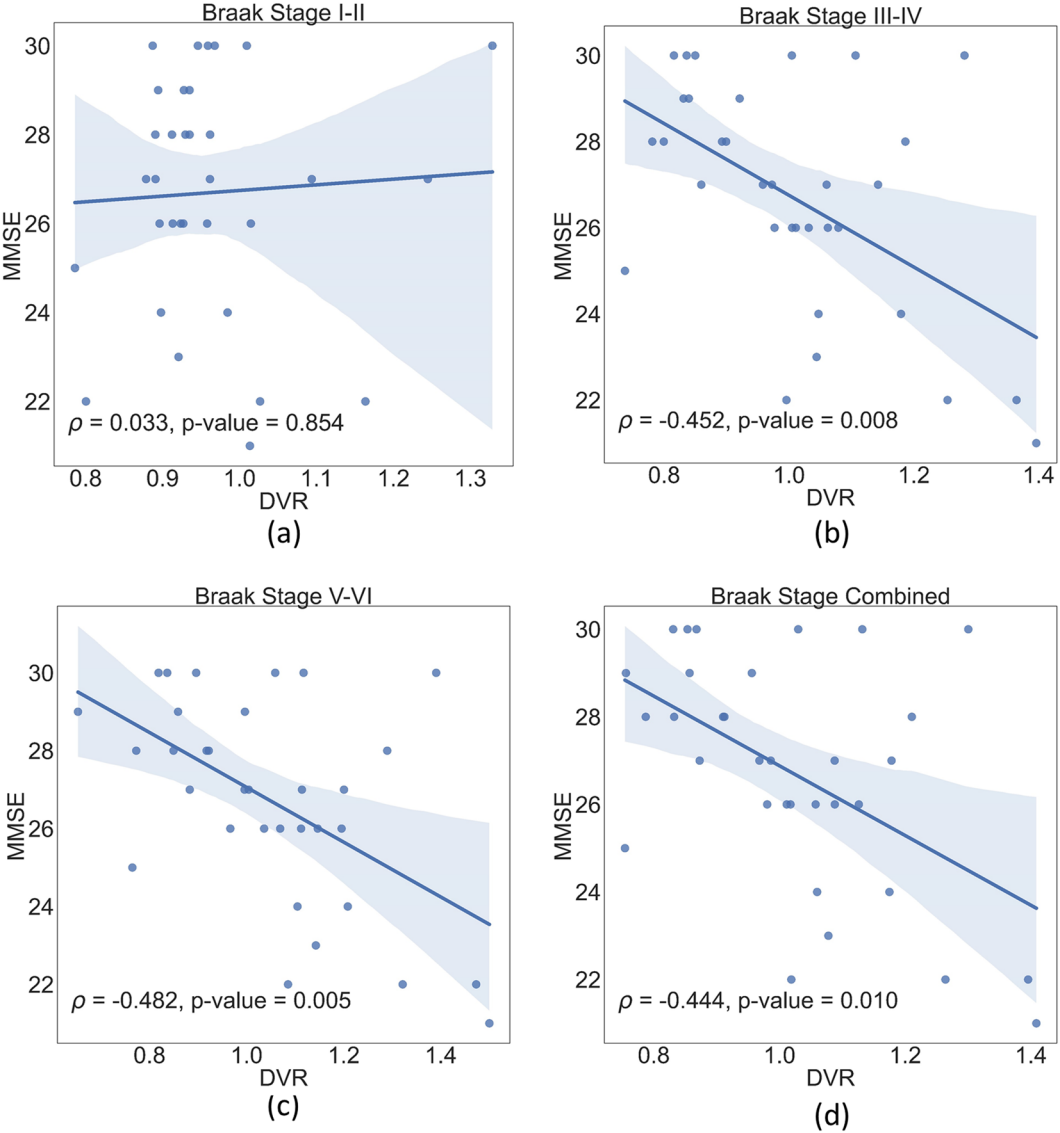
DVR of various Braak stages correlate with MMSE in MCI. Plot (**a**) has a weak positive correlation for Braak I–II, while (**b**–**d**) have a negative correlation for III–IV, V–VI, and all stages combined (FDR-*p* < 0.05). Linear fit (solid line) and 95% confidence interval (shadowed area) are shown

Spearman’s correlation (*ρ*) was computed between DVR and MMSE in MCI and controls (Fig. 4). Correlation was weak in early Braak stages I–II (*ρ* = 0.064; FDR-*p* = 0.628), whereas stronger and highly significant correlation was found in later Braak stages: III–IV (*ρ* = − 0.424; FDR-*p* = 0.001), V–VI (*ρ* = − 0.440; FDR-*p* = 0.0004), and all stages combined (*ρ* = − 0.430; FDR-*p* = 0.001). Figure 5 shows correlation only in MCI, where DVR and MMSE associations were stronger in later Braak stages: I–II (*ρ* = 0.033; FDR-*p* = 0.854), III–IV (ρ = − 0.452; FDR-*p* = 0.008), V–VI (*ρ* = − 0.482; FDR-*p* = 0.005), and all stages combined (*ρ* = − 0.444; FDR-*p* = 0.010).

### ML-based MMSE predictions

**Fig. 6:**
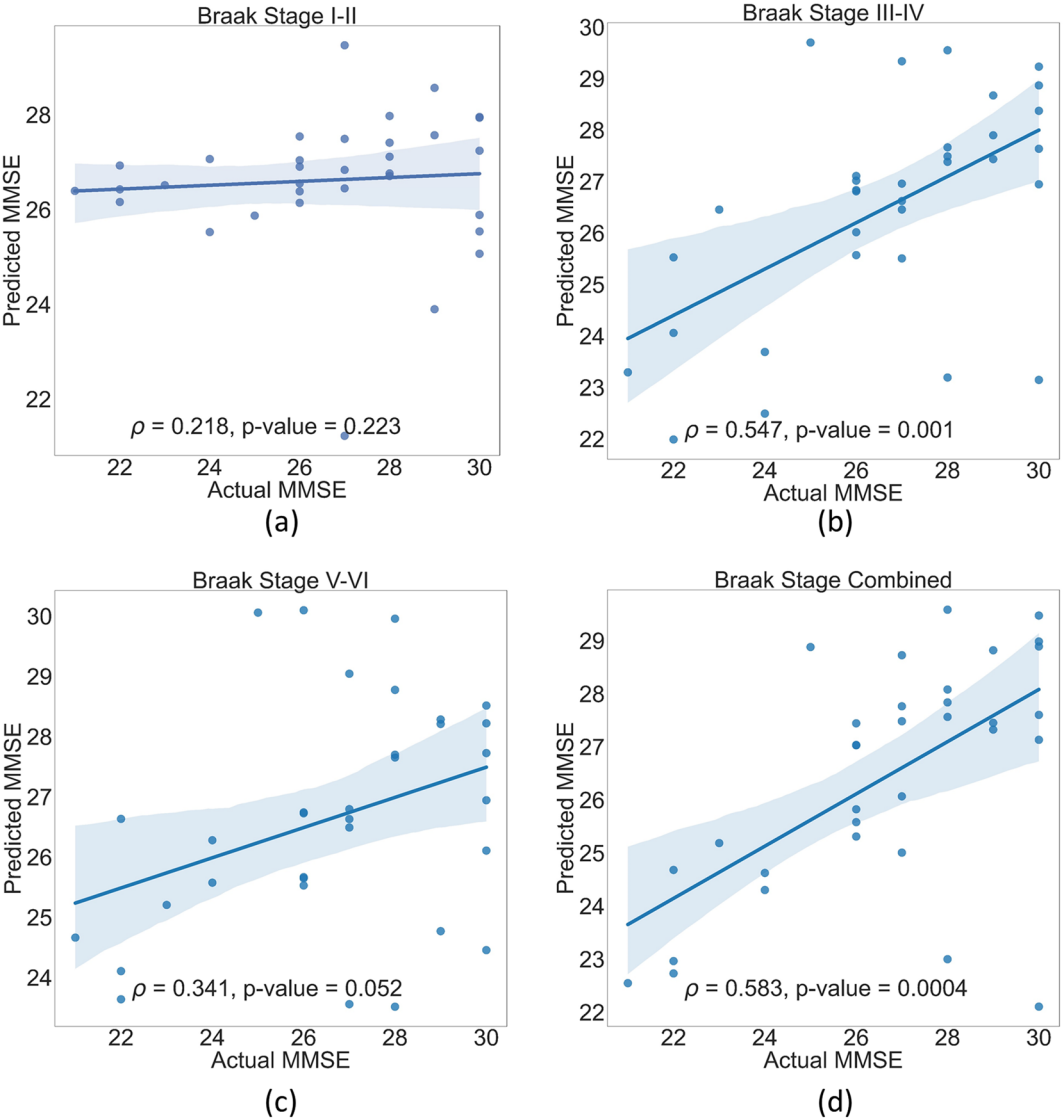
Positive correlation between predicted and actual MMSE in MCI using SVR modeling. While the correlation in (**a**) was weak, there was statistical significance (FDR-*p* < 0.05) in (**b**–**d**), especially all stages combined (**d**) had a stronger correlation. The linear fit (solid line) and 95% confidence interval (shadowed area) are shown

**Fig. 7:**
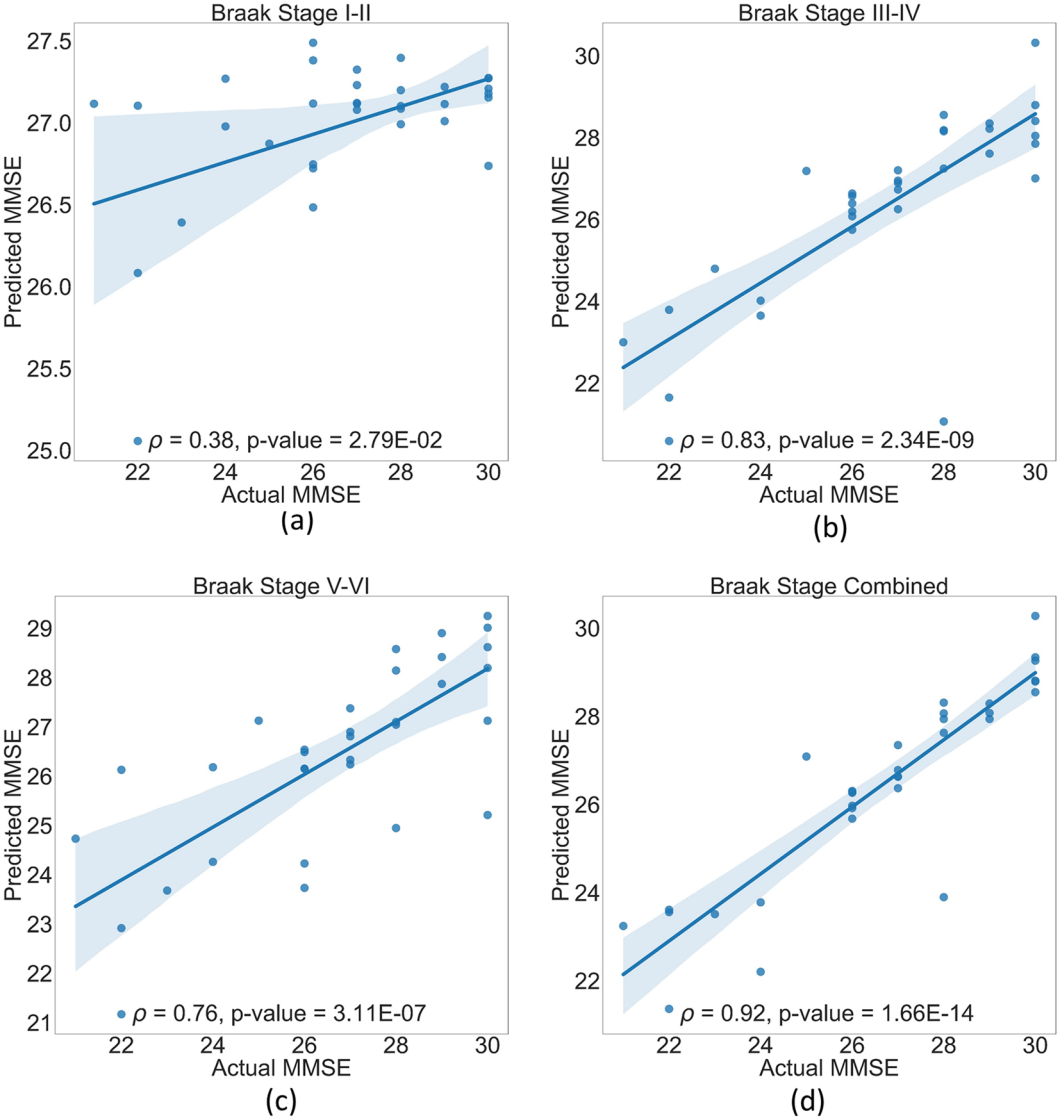
Positive correlation between predicted and actual MMSE in MCI using ANN modeling. While the correlation in (**a**) was weak, there was statistical significance (FDR-p < 0.05) in (**b**–**d**), especially all stages combined (**d**) had a stronger correlation. The linear fit (solid line) and 95% confidence interval (shadowed area) are shown

We tested whether regional DVR values could predict MMSE in MCI patients at different Braak Stages using our SVR and ANN models. Using SVR, the predicted and actual MMSE in MCI did not significantly correlate for Braak Stages I–II (*ρ* = 0.069; FDR-*p* = 0.218) (Fig. 6a). However, the correlation was higher for Braak Stages: III–IV (*ρ* = 0.547; FDR-*p* = 0.001), V–VI (*ρ* = 0.341; FDR-*p* = 0.052), and all stages combined (*ρ* = 0.583; FDR-*p* = 0.0004) (panels b–d). Using ANN (Fig. 7), the predicted and actual MMSE in MCI significantly correlated in Braak Stages: I–II (*ρ* = 0.383; FDR-*p* = 0.0028), III–IV (*ρ* = 0.830; FDR-*p* = 2.34E−9), V–VI (*ρ* = 0.759; FDR-*p* = 3.11E−7), and all stages combined (*ρ* = 0.924; FDR-*p* = 1.66E−14) (panels a–d). The superiority of ANN over SVR is confirmed by the average training and test loss of the SVR and ANN models throughout all 10 folds of data. The average training and test loss of the SVR and ANN models for each Braak stage is presented in Table 3. It would not be unreasonable to claim that when implementing Braak staging DVR to predict MMSE, the ANN model outperformed the SVR.

**Table 3:** Training and test losses of the SVR and ANN models for each Braak stage regions

Braak regions	SVR model		ANN model	
	Training	Test	Training	Test
I–II	1.47	2.24	1.94	2.02
III–IV	1.18	1.66	1.03	1.06
V–VI	1.35	2.12	1.22	1.34
Combined	1.01	1.53	0.76	0.83

### Significant MCI predictors

**Fig. 8:**
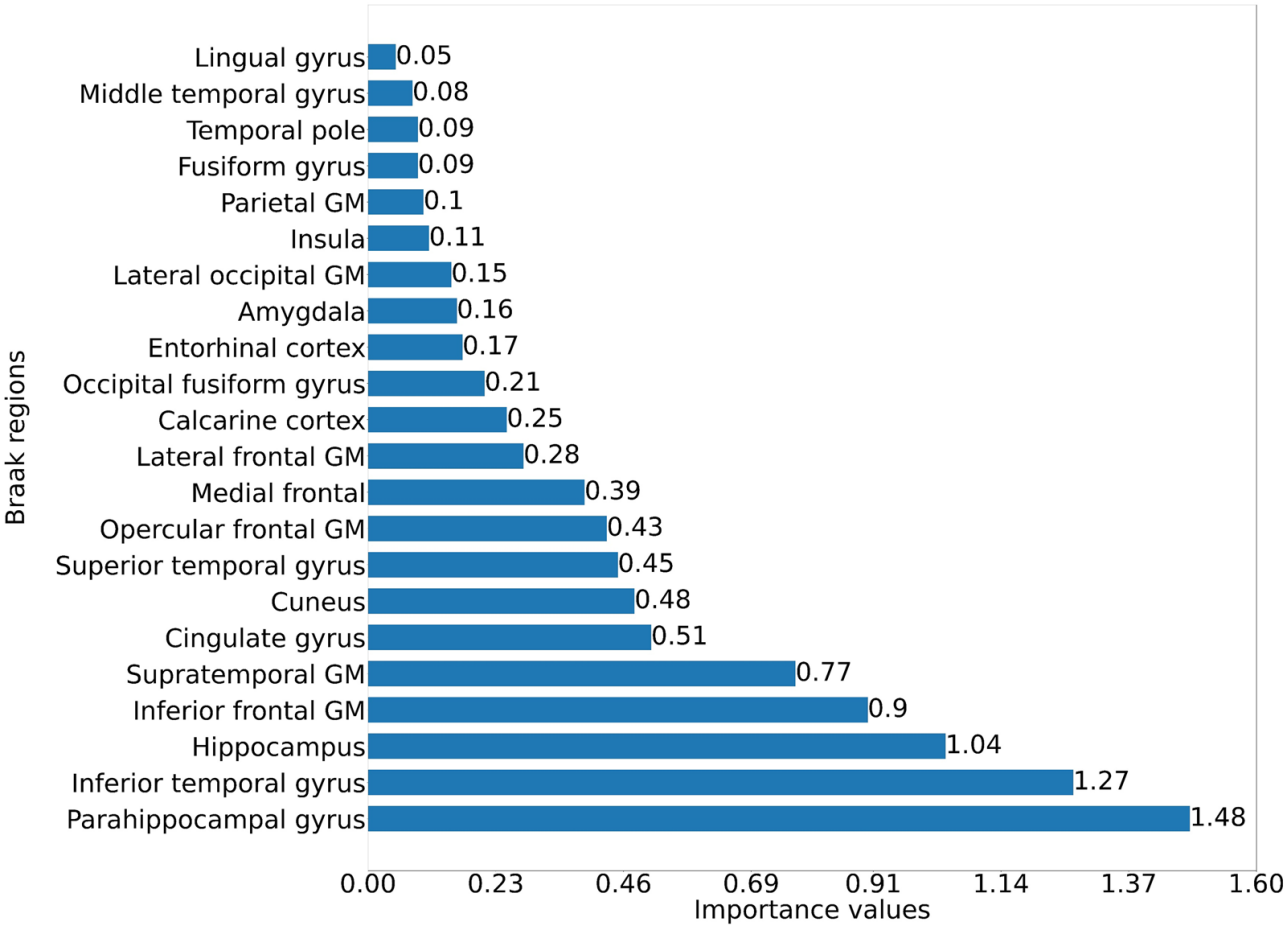
Chart compares the feature importance of the ANN model using the LIME method for different Braak regions. The importance values for each region are displayed on the *x*-axis, with the labels of the respective brain regions on the *y*-axis

We opted to focus only on the ANN model to compute feature significance, because the SVR model failed to perform well in predicting MMSE and had a smaller correlation between actual and predicted MMSE, as shown in Fig. 6. The ANN model demonstrated a higher correlation between actual and predicted MMSE, particularly when all Braak regions were included, as shown in Fig. 7. Furthermore, because some Braak regions have fewer traits than others, it would be good to determine whether specific regions are crucial across all Braak regions. When all Braak regions are combined, the parahippocampal gyrus is the most significant MCI predictor, followed by the inferior temporal gyrus, hippocampus, inferior frontal GM, and supratemporal GM, as shown in Fig. 8.

## Discussion

In this study, we proposed a novel multi-folded analytical framework demonstrating the multivariate relationships between Aβ DVR and cognition. We took Aβ DVR and cognition from the same individuals, trained SVR and ANN models on those data using standard CV techniques, and predicted cognition from Aβ DVR alone. Our analyses suggest that MCI had higher DVRs in later stages, strongly correlating to cognitive decline in stages III–IV and VI–V. When LIME and ANN were combined, the parahippocampal gyrus, inferior temporal gyrus, and hippocampus were the most dominant Aβ regional hubs for predicting cognitive decline. This suggests that these regions may play a key role in early AD mechanisms. Our approach is crucial for Aβ biomarker research for early detection of cognitive decline and aids in advancing computer-assisted diagnostic ML approaches. These approaches are different, and the results are promising compared to the established Aβ analysis approaches and related patterns [9, 81].

### Regional Aβ accumulation as a sensitive biomarker for disease progression

The clear difference between DVR in controls and MCI at different Braak stages supports our hypothesis that regional Aβ accumulation is a critical and sensitive biomarker for disease progression compared to other established biomarkers [82]. The effect of neuropathological changes on cognitive decline in MCI is complex, and the transition from normal cognition to MCI is complicated by several factors, including age, genetic predisposition [11, 83], and cognitive reserve [84]. It is well-established that the hippocampus and entorhinal cortex, which represent the topographic areas of Braak stages I–II, are the first to show significant alterations with cognitive decline in the form of neurofibrillary tangles [14, 85, 86] and atrophic changes [87]. The hippocampus and its impaired functional roles with other brain regions are widely reported in MCI [88]. Other brain regions in the Braak stages have also shown broad functional relationships with cognition in health and MCI [89–93].

### ML analysis of Aβ accumulation and cognitive function

The significant correlation between MMSE and DVR, especially in later Braak stages III–VI of MCI, further supports the hypothesis that more Aβ accumulation leads to worse cognitive outcomes, eventually leading to a higher risk of developing AD dementia. This correlation might also help clarify the relationships between Aβ accumulation and tau and their mutual effect on cognitive abilities and disease progression in later stages of the disease, i.e., symptomatic AD. The correlation between DVR and MMSE was weak in the early Braak stage I–II spatial distribution. Surprisingly, controls showed slightly higher DVR than MCI at this stage, albeit with a small effect size. This finding may be attributed to some controls being on the borderline of meeting the clinical diagnosis of MCI or transitioning to the MCI phase. Using ANN and SVR models, we reliably predicted cognitive impairment in the MCI population starting at Braak stages III–IV. The success of these models in predicting MMSE based on DVR at different Braak stages is a highly promising proof-of-concept approach. These results highlight the significance of ML algorithms in understanding and diagnosing AD pathophysiology, which supports previously published work on the reliability of ML models [94, 95].

### ML-based feature importance

Using the combination of ANN and LIME methods, we determined the significance of Braak staging brain regions, and it was found that the parahippocampal gyrus, inferior temporal gyrus, and hippocampus Aβ biomarkers were the top three significant features for predicting cognitive dysfunction. These findings broadly align with previous research indicating that alteration in the parahippocampal gyrus and hippocampus is an early biomarker of AD [96–100]. The inferior temporal gyrus region has been also previously linked with MCI or early AD [101–104]. To determine the most reliable and robust regional Aβ features for precise MCI prediction, assessing the feature importance across multiple models and data sets is crucial, especially with larger data sets.

### Limitations and future work

ML approaches typically require larger sample sizes for robust results. However, with our proof-of-concept approach, we were able to generate clear predictions and outcomes with a relatively small sample size. As a result, we anticipate strong findings in future studies with larger sample sizes. While using ML algorithms in only Braak staging regions may limit the generalization of Aβ accumulation as a predictor of cognitive abilities, combining other regions can enhance the ability to predict cognitive dysfunction in individuals with MCI and other populations. By incorporating additional brain regions, such as those involved in memory and executive functioning, we may improve our predictive models’ accuracy and robustness. The PET scans we studied were obtained from only two sites. The future studies should also focus on considering a larger number of features, including voxel measures, and associated dimensional reduction techniques, and studying tau PET and other imaging modalities from a larger number of sites. These approaches might increase the reliability of the findings from multiple aspects of AD mechanisms.

## Conclusion

Our findings demonstrate a significant difference in Aβ accumulation between MCI and controls, particularly in the later stages of the spatial distribution of Braak stages. The strong association between Aβ DVR and cognitive dysfunction highlights the importance of Aβ as a biomarker for MCI. The most important Braak regions associated with cognitive impairment were carefully identified by combining LIME feature significance and ANN techniques. This integration offers a promising strategy for comprehending the links between regional Aβ biomarkers and cognitive impairment. Our approach highlights the ML model’s potential in conjunction with the feature importance attributes in enhancing biomarker identification and suggests that further research utilizing these tools may lead to earlier diagnosis and intervention in the progression of MCI and related disorders.

## Data Availability

The data sets used and/or analyzed during the current study are available from the corresponding author upon reasonable request.

## References

[1] Deschaintre Y, Richard F, Leys D, Pasquier F (2009). Treatment of vascular risk factors is associated with slower decline in Alzheimer disease. Neurology.

[2] Fleisher AS, Chen K, Quiroz YT, Jakimovich LJ, Gutierrez Gomez M, Langois CM, Langbaum JBS, Roontiva A, Thiyyagura P, Lee W, Ayutyanont N, Lopez L, Moreno S, Muñoz C, Tirado V, Acosta-Baena N, Fagan AM, Giraldo M, Garcia G (2015). Associations between biomarkers and age in the presenilin 1 E280A autosomal dominant Alzheimer disease kindred: a cross-sectional study. JAMA Neurol.

[3] Petersen RC, Doody R, Kurz A, Mohs RC, Morris JC, Rabins PV, Ritchie K, Rossor M, Thal L, Winblad B (2001). Current concepts in mild cognitive impairment. Arch Neurol.

[4] Reitz C (2012). Alzheimer's disease and the amyloid cascade hypothesis: a critical review. Int J Alzheimer’s Dis.

[5] Wu T, Lin D, Cheng Y, Jiang S, Riaz MW, Fu N, Mou C, Ye M, Zheng Y (2022). Amyloid cascade hypothesis for the treatment of Alzheimer's disease: progress and challenges. Aging Dis.

[6] Hardy JA, Higgins GA (1992). Alzheimer's disease: the amyloid cascade hypothesis. Science.

[7] Musiek ES, Holtzman DM (2015). Three dimensions of the amyloid hypothesis: time, space and 'wingmen'. Nat Neurosci.

[8] Cohen AD, Klunk WE (2014). Early detection of Alzheimer's disease using PiB and FDG PET. Neurobiol Dis.

[9] Klunk WE, Engler H, Nordberg A, Wang Y, Blomqvist G, Holt DP, Bergström M, Savitcheva I, Huang G-F, Estrada S, Ausén B, Debnath ML, Barletta J, Price JC, Sandell J, Lopresti BJ, Wall A, Koivisto P, Antoni G (2004). Imaging brain amyloid in Alzheimer's disease with Pittsburgh Compound-B. Ann Neurol.

[10] Ingelsson M, Fukumoto H, Newell K, Growdon J, Hedley-Whyte E, Frosch M, Albert M, Hyman B, Irizarry M (2004). Early Aβ accumulation and progressive synaptic loss, gliosis, and tangle formation in AD brain. Neurology.

[11] Stephan BCM, Hunter S, Harris D, Llewellyn DJ, Siervo M, Matthews FE, Brayne C (2012). The neuropathological profile of mild cognitive impairment (MCI): a systematic review. Mol Psychiatry.

[12] Blennow K, Zetterberg H (2018). Biomarkers for Alzheimer's disease: current status and prospects for the future. J Intern Med.

[13] Zetterberg H, Bendlin BB (2021). Biomarkers for Alzheimer's disease-preparing for a new era of disease-modifying therapies. Mol Psychiatry.

[14] Braak H, Braak E (1991). Neuropathological stageing of Alzheimer-related changes. Acta Neuropathol.

[15] Kim M, Kim SJ, Park JE, Yun J, Shim WH, Oh JS, Oh M, Roh JH, Seo SW, Oh SJ, Kim JS (2021). Combination of automated brain volumetry on MRI and quantitative tau deposition on THK-5351 PET to support diagnosis of Alzheimer’s disease. Sci Rep.

[16] Bennett DA, Schneider JA, Wilson RS, Bienias JL, Arnold SE (2004). Neurofibrillary tangles mediate the association of amyloid load with clinical alzheimer disease and level of cognitive function. Arch Neurol.

[17] Biel D, Brendel M, Rubinski A, Buerger K, Janowitz D, Dichgans M, Franzmeier N, Alzheimer’s Disease Neuroimaging, I.  (2021). Tau-PET and in vivo Braak-staging as prognostic markers of future cognitive decline in cognitively normal to demented individuals. Alzheimer's Res Ther.

[18] Bucci M, Chiotis K, Nordberg A, for the Alzheimer’s Disease Neuroimaging, I.  (2021). Alzheimer’s disease profiled by fluid and imaging markers: tau PET best predicts cognitive decline. Mol Psychiatry.

[19] Giannakopoulos P, Herrmann FR, Bussiere T, Bouras C, Kovari E, Perl DP, Morrison JH, Gold G, Hof PR (2003). Tangle and neuron numbers, but not amyloid load, predict cognitive status in Alzheimer's disease. Neurology.

[20] Ossenkoppele R, Schonhaut DR, Schöll M, Lockhart SN, Ayakta N, Baker SL, O'Neil JP, Janabi M, Lazaris A, Cantwell A, Vogel J, Santos M, Miller ZA, Bettcher BM, Vossel KA, Kramer JH, Gorno-Tempini ML, Miller BL, Jagust WJ, Rabinovici GD (2016). Tau PET patterns mirror clinical and neuroanatomical variability in Alzheimer's disease. Brain A J Neurol.

[21] Wang L, Benzinger TL, Su Y, Christensen J, Friedrichsen K, Aldea P, McConathy J, Cairns NJ, Fagan AM, Morris JC, Ances BM (2016). Evaluation of tau imaging in staging alzheimer disease and revealing interactions between β-amyloid and tauopathy. JAMA Neurol.

[22] Jack CR, Wiste HJ, Botha H, Weigand SD, Therneau TM, Knopman DS, Graff-Radford J, Jones DT, Ferman TJ, Boeve BF, Kantarci K, Lowe VJ, Vemuri P, Mielke MM, Fields JA, Machulda MM, Schwarz CG, Senjem ML, Gunter JL, Petersen RC (2019). The bivariate distribution of amyloid-β and tau: relationship with established neurocognitive clinical syndromes. Brain J Neurol.

[23] Lockhart SN, Schöll M, Baker SL, Ayakta N, Swinnerton KN, Bell RK, Mellinger TJ, Shah VD, O'Neil JP, Janabi M, Jagust WJ (2017). Amyloid and tau PET demonstrate region-specific associations in normal older people. Neuroimage.

[24] Chui HC, Zarow C, Mack WJ, Ellis WG, Zheng L, Jagust WJ, Mungas D, Reed BR, Kramer JH, DeCarli CC (2006). Cognitive impact of subcortical vascular and Alzheimer's disease pathology. Ann Neurol.

[25] Rosenberger AF, Hilhorst R, Coart E, García Barrado L, Naji F, Rozemuller AJ, van der Flier WM, Scheltens P, Hoozemans JJ, van der Vies SM (2016). Protein kinase activity decreases with higher Braak stages of Alzheimer’s disease pathology. J Alzheimers Dis.

[26] Jack CR, Knopman DS, Jagust WJ, Shaw LM, Aisen PS, Weiner MW, Petersen RC, Trojanowski JQ (2010). Hypothetical model of dynamic biomarkers of the Alzheimer's pathological cascade. Lancet Neurol.

[27] LaMontagne PJ, Benzinger TLS, Morris JC, Keefe S, Hornbeck R, Xiong C, Grant E, Hassenstab J, Moulder K, Vlassenko AG, Raichle ME, Cruchaga C, Marcus D (2019). OASIS-3: longitudinal neuroimaging, clinical, and cognitive dataset for normal aging and Alzheimer disease.

[28] Chand GB, Thakuri DS, Soni B (2022). Salience network anatomical and molecular markers are linked with cognitive dysfunction in mild cognitive impairment. J Neuroimaging.

[29] Chand GB, Singhal P, Dwyer DB, Wen J, Erus G, Doshi J, Srinivasan D, Mamourian E, Varol E, Sotiras A, Hwang G, Dazzan P, Kahn RS, Schnack HG, Zanetti MV, Meisenzahl E, Busatto GF, Crespo-Facorro B, Pantelis C (2022). Schizophrenia imaging signatures and their associations with cognition, psychopathology, and genetics in the general population. Am J Psychiatry.

[30] Marcus DS, Fotenos AF, Csernansky JG, Morris JC, Buckner RL (2010). Open access series of imaging studies: longitudinal MRI data in nondemented and demented older adults. J Cogn Neurosci.

[31] Morris JC (1993). The clinical dementia rating (CDR). Neurology.

[32] Morris JC, Storandt M, Miller JP, McKeel DW, Price JL, Rubin EH, Berg L (2001). Mild cognitive impairment represents early-stage Alzheimer disease. Arch Neurol.

[33] Folstein MF, Folstein SE, McHugh PR (1975). Mini-mental state. J Psychiatr Res.

[34] Diniz BSO, Yassuda MS, Nunes PV, Radanovic M, Forlenza OV (2007). Mini-mental State Examination performance in mild cognitive impairment subtypes. Int Psychogeriatr.

[35] Mitchell AJ (2009). A meta-analysis of the accuracy of the mini-mental state examination in the detection of dementia and mild cognitive impairment. J Psychiatric Res.

[36] Shulman KI, Herrmann N, Brodaty H, Chiu H, Lawlor B, Ritchie K, Scanlan JM (2006). IPA survey of brief cognitive screening instruments. Int Psychogeriatr.

[37] Choe YM, Lee BC, Choi IG, Suh GH, Lee DY, Kim JW (2020). MMSE subscale scores as useful predictors of AD conversion in mild cognitive impairment. Neuropsychiatr Dis Treat.

[38] Patnode CD, Perdue LA, Rossom RC, Rushkin MC, Redmond N, Thomas RG, Lin JS (2020). Screening for cognitive impairment in older adults. JAMA.

[39] Su Y, Dong J, Sun J, Zhang Y, Ma S, Li M, Zhang A, Cheng B, Cai S, Bao Q, Wang S, Zhu P (2021). Cognitive function assessed by Mini-mental state examination and risk of all-cause mortality: a community-based prospective cohort study. BMC Geriatr.

[40] Chand GB, Dwyer DB, Erus G, Sotiras A, Varol E, Srinivasan D, Doshi J, Pomponio R, Pigoni A, Dazzan P, Kahn RS, Schnack HG, Zanetti MV, Meisenzahl E, Busatto GF, Crespo-Facorro B, Pantelis C, Wood SJ, Zhuo C (2020). Two distinct neuroanatomical subtypes of schizophrenia revealed using machine learning. Brain J Neurol.

[41] Chand GB, Habes M, Dolui S, Detre JA, Wolk DA, Davatzikos C (2020). Estimating regional cerebral blood flow using resting-state functional MRI via machine learning. J Neurosci Methods.

[42] Doshi J, Erus G, Ou Y, Resnick SM, Gur RC, Gur RE, Satterthwaite TD, Furth S, Davatzikos C, Alzheimer's Neuroimaging I (2016). MUSE: MUlti-atlas region Segmentation utilizing Ensembles of registration algorithms and parameters, and locally optimal atlas selection. Neuroimage.

[43] Logan J, Volkow ND, Fowler JS, Wang GJ, Dewey SL, MacGregor R, Schlyer D, Gatley SJ, Pappas N, King P (1994). Effects of blood flow on [11C]raclopride binding in the brain: model simulations and kinetic analysis of PET data. J Cereb Blood Flow Metab.

[44] Moses WW (2011). Fundamental limits of spatial resolution in PET. Nucl Instr Methods Phys Res Sect A Accel Spectr Detect Assoc Equip.

[45] Zhu Y, Bilgel M, Gao Y, Rousset OG, Resnick SM, Wong DF, Rahmim A (2021). Deconvolution-based partial volume correction of PET images with parallel level set regularization. Phys Med Biol.

[46] Boser BE, Guyon IM, Vapnik VN (1992) A training algorithm for optimal margin classifiers. In: Proceedings of the fifth annual workshop on Computational learning theory, Pittsburgh, Pennsylvania, USA. 10.1145/130385.130401

[47] Cristianini N, Shawe-Taylor J (2000). An introduction to support vector machines and other kernel-based learning methods.

[48] Noble WS (2006). What is a support vector machine?. Nat Biotechnol.

[49] LeCun Y, Bengio Y, Hinton G (2015). Deep learning. Nature.

[50] Rusk N (2016). Deep learning. Nat Methods.

[51] Jain AK, Jianchang M, Mohiuddin KM (1996). Artificial neural networks: a tutorial. Computer.

[52] Krogh A (2008). What are artificial neural networks?. Nat Biotechnol.

[53] Abraham A, Pedregosa F, Eickenberg M, Gervais P, Mueller A, Kossaifi J, Gramfort A, Thirion B, Varoquaux G (2014). Machine learning for neuroimaging with scikit-learn. Front Neuroinform.

[54] Pedregosa F, Varoquaux G, Gramfort A, Michel V, Thirion B, Grisel O, Blondel M, Prettenhofer P, Weiss R, Dubourg V, Vanderplas J, Passos A, Cournapeau D, Brucher M, Perrot M, Duchesnay E (2011). Scikit-learn: machine learning in python. J Mach Learn Res.

[55] Vert J-P, Tsuda K, Schlkopf B (2004). A primer on kernel methods. Kernel Methods Comput Biol.

[56] Abadi M, Agarwal A, Barham P, Brevdo E, Chen Z, Citro C, Corrado GS, Davis A, Dean J, Devin M, Ghemawat S, Goodfellow I, Harp A, Irving G, Isard M, Jia Y, Jozefowicz R, Kaiser L, Kudlur M, . . . Zheng X (2016) TensorFlow: large-scale machine learning on heterogeneous distributed systems. arXiv:1603.04467. https://ui.adsabs.harvard.edu/abs/2016arXiv160304467A

[57] Chollet, F. (2015). Chollet, F. (2015) keras, GitHub. - References - Scientific Research Publishing. https://www.scirp.org/(S(351jmbntvnsjt1aadkposzje))/reference/ReferencesPapers.aspx?ReferenceID=1887532

[58] Hinton GE, Srivastava N, Krizhevsky A, Sutskever I, Salakhutdinov RR (2012) Improving neural networks by preventing co-adaptation of feature detectors. In: arXiv.

[59] Srivastava N, Hinton G, Krizhevsky A, Sutskever I, Salakhutdinov R (2014). Dropout: a simple way to prevent neural networks from overfitting. J Mach Learn Res.

[60] Fukushima K (1969). Visual feature extraction by a multilayered network of analog threshold elements. IEEE Trans Syst Sci Cybern.

[61] Fukushima K, Miyake S (1982). Neocognitron: a self-organizing neural network model for a mechanism of visual pattern recognition. Competition and cooperation in neural nets.

[62] Glorot X, Bordes A, Bengio Y (2011) Deep sparse rectifier neural networks. In: International conference on artificial intelligence and statistics.

[63] Kingma DP, Ba J (2014) Adam: a method for stochastic optimization. arXiv preprint arXiv:1412.6980.

[64] Hunter JD (2007). Matplotlib: a 2D graphics environment. Comput Sci Eng.

[65] Waskom M (2021). seaborn: statistical data visualization. J Open Sour Softw.

[66] Bzdok D, Ioannidis JP (2019). Exploration, inference, and prediction in neuroscience and biomedicine. Trends Neurosci.

[67] Davatzikos C (2019). Machine learning in neuroimaging: progress and challenges. Neuroimage.

[68] Ribeiro MT, Singh S, Guestrin C (2016) “Why should i trust you?” Explaining the predictions of any classifier. In: Proceedings of the 22nd ACM SIGKDD international conference on knowledge discovery and data mining.

[69] Linardatos P, Papastefanopoulos V, Kotsiantis S (2020). Explainable ai: a review of machine learning interpretability methods. Entropy.

[70] Ribeiro MT, Singh S, Guestrin C. https://github.com/marcotcr/lime

[71] Lundberg SM, Lee SI (2017) A unified approach to interpreting model predictions. Advances in neural information processing systems. p. 30.

[72] Tibshirani R (1996). Regression shrinkage and selection via the lasso. J Roy Stat Soc: Ser B (Methodol).

[73] Jansen T, Geleijnse G, Van Maaren M, Hendriks MP, Ten Teije A, Moncada-Torres A (2020). Machine learning explainability in breast cancer survival. Digital personalized health and medicine.

[74] Man X, Chan EP (2021). The best way to select features? Comparing mda, lime, and shap. J Fin Data Sci.

[75] Zafar MR, Khan NM (2019) DLIME: A deterministic local interpretable model-agnostic explanations approach for computer-aided diagnosis systems. arXiv preprint arXiv:1906.10263.

[76] Zhang Y, Song K, Sun Y, Tan S, Udell M (2019) “Why should you trust my explanation?” Understanding uncertainty in LIME explanations. arXiv preprint arXiv:1904.12991.

[77] Ilay AA, Painsky A (2021) Feature importance in gradient boosting trees with cross-validation feature selection. arXiv e-prints, arXiv: 2109.05468. 10.3390/e24050687 PMC9140774 35626570

[78] Fay MP, Proschan MA (2010). Wilcoxon-Mann-Whitney or t-test? On assumptions for hypothesis tests and multiple interpretations of decision rules. Stat Surv.

[79] Virtanen P, Gommers R, Oliphant TE, Haberland M, Reddy T, Cournapeau D, Burovski E, Peterson P, Weckesser W, Bright J (2020). SciPy 1.0: fundamental algorithms for scientific computing in Python. Nat Methods.

[80] Benjamini Y, Hochberg Y (1995). Controlling the false discovery rate: a practical and powerful approach to multiple testing. J R Stat Soc Ser B Methodol.

[81] Thal DR, Rüb U, Orantes M, Braak H (2002). Phases of Aβ-deposition in the human brain and its relevance for the development of AD. Neurology.

[82] Pfeil J, Hoenig MC, Doering E, van Eimeren T, Drzezga A, Bischof GN, Alzheimer's Disease Neuroimaging, I (2021). Unique regional patterns of amyloid burden predict progression to prodromal and clinical stages of Alzheimer's disease. Neurobiol Aging.

[83] Mufson EJ, Malek-Ahmadi M, Snyder N, Ausdemore J, Chen K, Perez SE (2016). Braak stage and trajectory of cognitive decline in noncognitively impaired elders. Neurobiol Aging.

[84] Mazzeo S, Padiglioni S, Bagnoli S, Bracco L, Nacmias B, Sorbi S, Bessi V (2019). The dual role of cognitive reserve in subjective cognitive decline and mild cognitive impairment: a 7-year follow-up study. J Neurol.

[85] Van Hoesen GW, Hyman BT (1990). Hippocampal formation: anatomy and the patterns of pathology in Alzheimer's disease. Prog Brain Res.

[86] Van Hoesen GW, Hyman BT, Damasio AR (1991). Entorhinal cortex pathology in Alzheimer's disease. Hippocampus.

[87] Pennanen C, Kivipelto M, Tuomainen S, Hartikainen P, Hänninen T, Laakso MP, Hallikainen M, Vanhanen M, Nissinen A, Helkala E-L, Vainio P, Vanninen R, Partanen K, Soininen H (2004). Hippocampus and entorhinal cortex in mild cognitive impairment and early AD. Neurobiol Aging.

[88] Chand GB, Hajjar I, Qiu D (2018). Disrupted interactions among the hippocampal, dorsal attention, and central-executive networks in amnestic mild cognitive impairment. Hum Brain Mapp.

[89] Aschenbrenner AJ, Gordon BA, Benzinger TLS, Morris JC, Hassenstab JJ (2018). Influence of tau PET, amyloid PET, and hippocampal volume on cognition in Alzheimer disease. Neurology.

[90] Chand GB, Dhamala M (2016). The salience network dynamics in perceptual decision-making. Neuroimage.

[91] Chand GB, Wu J, Hajjar I, Qiu D (2017). Interactions of the salience network and its subsystems with the default-mode and the central-executive networks in normal aging and mild cognitive impairment. Brain Connectivity.

[92] Chand GB, Wu J, Qiu D, Hajjar I (2017). Racial differences in insular connectivity and thickness and related cognitive impairment in hypertension. Front Aging Neurosci.

[93] Timmers T, Ossenkoppele R, Verfaillie SCJ, van der Weijden CWJ, Slot RER, Wesselman LMP, Windhorst AD, Wolters EE, Yaqub M, Prins ND, Lammertsma AA, Scheltens P, van der Flier WM, van Berckel BNM (2019). Amyloid PET and cognitive decline in cognitively normal individuals: the SCIENCe project. Neurobiol Aging.

[94] Gill S, Mouches P, Hu S, Rajashekar D, MacMaster FP, Smith EE, Forkert ND, Ismail Z, Alzheimer’s Disease Neuroimaging, I.  (2020). Using machine learning to predict dementia from neuropsychiatric symptom and neuroimaging data. J Alzheimer's Dis JAD.

[95] James C, Ranson JM, Everson R, Llewellyn DJ (2021). Performance of machine learning algorithms for predicting progression to dementia in memory clinic patients. JAMA Netw Open.

[96] Chua TC, Wen W, Slavin MJ, Sachdev PS (2008). Diffusion tensor imaging in mild cognitive impairment and Alzheimer's disease: a review. Curr Opin Neurol.

[97] Echávarri C, Aalten P, Uylings HB, Jacobs H, Visser PJ, Gronenschild E, Verhey F, Burgmans S (2011). Atrophy in the parahippocampal gyrus as an early biomarker of Alzheimer’s disease. Brain Struct Funct.

[98] Kesslak JP, Nalcioglu O, Cotman CW (1991). Quantification of magnetic resonance scans for hippocampal and parahippocampal atrophy in Alzheimer's disease. Neurology.

[99] Magnin B, Mesrob L, Kinkingnhun S, Plgrini-Issac M, Colliot O, Sarazin M, Dubois B, Lehricy S, Benali H (2009). Support vector machine-based classification of Alzheimer’s disease from whole-brain anatomical MRI. Neuroradiology.

[100] van Hoesen GW, Augustinack JC, Dierking J, Redman SJ, Thangavel R (2000). The parahippocampal gyrus in Alzheimer's disease: clinical and preclinical neuroanatomical correlates. Ann N Y Acad Sci.

[101] Beach TG, Kuo Y-M, Spiegel K, Emmerling MR, Sue LI, Kokjohn K, Roher AE (2000). The cholinergic deficit coincides with Aβ deposition at the earliest histopathologic stages of Alzheimer disease. J Neuropathol Exp Neurol.

[102] Convit A, De Asis J, De Leon M, Tarshish C, De Santi S, Rusinek H (2000). Atrophy of the medial occipitotemporal, inferior, and middle temporal gyri in non-demented elderly predict decline to Alzheimer’s disease☆. Neurobiol Aging.

[103] Liu X-C, Qi X-H, Fang H, Zhou K-Q, Wang Q-S, Chen G-H (2021). Increased MANF expression in the inferior temporal gyrus in patients with Alzheimer disease. Front Aging Neurosci.

[104] Scheff SW, Price DA, Schmitt FA, Scheff MA, Mufson EJ (2011). Synaptic loss in the inferior temporal gyrus in mild cognitive impairment and Alzheimer's disease. J Alzheimers Dis.

[105] Taha A, Soni B, Thakuri DS, Ritter E, Bhattarai P, Chand G (2022) Amyloid-beta biomarkers in Braak stages and their predictive relationships with cognitive impairment: support vector machine and deep learning approaches. bioRxiv. 2022–2009.

